# Waterlogging-induced changes in root architecture of germplasm accessions of the tropical forage grass *Brachiaria humidicola*

**DOI:** 10.1093/aobpla/plu017

**Published:** 2014-04-08

**Authors:** Juan Andrés Cardoso, Juan de la Cruz Jiménez, Idupulapati M. Rao

**Affiliations:** 1 Centro Internacional de Agricultura Tropical (CIAT), Apartado Aéreo 6713, Cali, Colombia; 2 Programa de doctorado Biología Agraria y Acuicultura, Universidad de Granada, Avenida de Fuente Nueva s/n, Granada 18071, Spain

**Keywords:** Lateral root proportion, oxygen deficiency, rooting depth, root length, soil flooding, vertical root distribution.

## Abstract

Waterlogging is one of the major factors limiting the productivity of pastures in the humid tropics. *Brachiaria humidicola* is a forage grass commonly used in zones prone to temporary waterlogging. *Brachiaria humidicola* accessions adapt to waterlogging by increasing aerenchyma in nodal roots above constitutive levels to improve oxygenation of root tissues. In some accessions, waterlogging reduces the number of lateral roots developed from main root axes. Waterlogging-induced reduction of lateral roots could be of adaptive value as lateral roots consume oxygen supplied from above ground via their parent root. However, a reduction in lateral root development could also be detrimental by decreasing the surface area for nutrient and water absorption. To examine the impact of waterlogging on lateral root development, an outdoor study was conducted to test differences in vertical root distribution (in terms of dry mass and length) and the proportion of lateral roots to the total root system (sum of nodal and lateral roots) down the soil profile under drained or waterlogged soil conditions. Plant material consisted of 12 *B. humidicola* accessions from the gene bank of the International Center for Tropical Agriculture, Colombia. Rooting depth was restricted by 21 days of waterlogging and confined to the first 30 cm below the soil surface. Although waterlogging reduced the overall proportion of lateral roots, its proportion significantly increased in the top 10 cm of the soil. This suggests that soil flooding increases lateral root proliferation of *B. humidicola* in the upper soil layers. This may compensate for the reduction of root surface area brought about by the restriction of root growth at depths below 30 cm. Further work is needed to test the relative efficiency of nodal and lateral roots for nutrient and water uptake under waterlogged soil conditions.

## Abstract (precis)

*Brachiaria humidicola*, a tropical forage grass, develops aerenchyma in nodal roots to adapt to waterlogging. A large body of work has focused on the functional role of aerenchyma in nodal roots under waterlogged soil conditions. On the other hand, quantification of responses of lateral roots to waterlogging has been often overlooked in past work. Our data indicated that although waterlogging reduced the overall proportion of lateral roots, its proportion significantly increased in the top 10 cm of the soil. This suggests that soil flooding increases lateral root proliferation of *B. humidicola* in upper soil layers. This may compensate the reduction of root surface area brought by the restriction of root growth at depths below 30 cm into waterlogged soil.

## Introduction

Daily news reflects the extremes in water availability around the world and modelling predicts that extreme events, including heavy precipitation, will increase in the future (Magrin *et al.* 2007; Allan and Soden 2008; O'Gorman and Scheneider 2009; Trenberth 2011). The term waterlogging is used to refer to flooding of the soil. This occurs when the infiltration of water from rainfall or flooding exceeds the rate of subsurface drainage and evapotranspiration (Bramley *et al.* 2011). Diffusion of gases, notably of oxygen, is ∼10 000 times slower in water than in air, leading to oxygen depletion in waterlogged soils as diffusive influx fails to keep pace with demand from root and microbial respiration (Elzenga and Veen 2010). Oxygen deficiency in the soil rooting zone might affect plant growth directly by limiting root aerobic respiration (Jackson 1985; Vartapetian and Jackson 1997). Many species well adapted to waterlogging have aerenchyma formed in root tissues that allows the internal transport of oxygen within the roots. This can at least partly compensate for oxygen shortage in the soil and support root aerobic respiration (Armstrong 1979; Colmer 2003).

A limitation for plant growth in tropical grasslands is temporary or permanent waterlogging (Baruch 1994). *Brachiaria humidicola* (Rendle) Schweickerdt is a stoloniferous perennial grass that grows well in areas of infertile acid and poorly drained soils subject to temporary waterlogging (Keller-Grein *et al.* 1996) and thus an important forage option under these conditions (Calisto *et al.* 2008). In a previous study, it was found that while *B. humidicola* developed aerenchyma in nodal roots even when well drained, aerenchyma increased further under waterlogging. This presumably allowed internally transported oxygen to sustain root aerobic respiration and elongation under soil oxygen shortage (Cardoso *et al.* 2013). The same study also found that lateral roots (i.e. roots developed from nodal roots) showed negligible aerenchyma and that a reduction in the number of lateral roots occurred in some *B. humidicola* accessions under waterlogged soil conditions (Cardoso *et al.* 2013). It has been proposed that a reduction in the number of lateral roots developed from an aerenchymatous root axis might be an advantage under oxygen-deficient conditions as lateral roots consume O_2_ from the aerenchyma of the parent root, thus decreasing O_2_ diffusion to the elongation zone of the parent root (Armstrong *et al.* 1983; Sorrell *et al.* 2000; Aguilar *et al.* 2003). However, reductions in lateral root development would, inevitably, reduce the surface area needed for nutrient and water absorption in waterlogged soil (Kirk 2003).

A large body of work has focused on the development and functional role of aerenchymatous roots under waterlogged soil conditions (Bailey-Serres and Voesenek 2008; Colmer and Voesenek 2009; Colmer and Greenway 2011; Yamauchi *et al.* 2013). On the other hand, despite being highly responsive to their environment, quantification of responses of lateral roots to waterlogging has been often overlooked in past work. Furthermore, there appears to be no published information regarding the relative contribution of the lateral root system (in dry mass or length) to the total root system in *B. humidicola* under any conditions. The present study is a follow-up to one showing significant reductions in root dry mass and penetration when 12 *B. humidicola* germplasm accessions were waterlogged (Cardoso *et al.* 2013). The main objective was to quantify differences in vertical root distribution (in terms of dry mass and length) and the contribution of the lateral root system (%) to the total root system (i.e. the sum of nodal and lateral roots) across soil depth. A detailed knowledge of morphological responses and intra-specific variation of *B. humidicola* will contribute to the development of efficient screening procedures for evaluating waterlogging tolerance of hybrids generated from the ongoing *Brachiaria* breeding programme of the International Center for Tropical Agriculture (CIAT).

## Methods

### Accessions and growing conditions

The methodology used in this study was similar to the one described by Cardoso *et al.* (2013). *Brachiaria humidicola* is a deep-rooted C_4_ grass of African origin. Twelve germplasm accessions of *B. humidicola* (CIAT 679, CIAT 6013, CIAT 6133, CIAT 6707, CIAT 16182, CIAT 16866, CIAT 16886, CIAT 16888, CIAT 26152, CIAT 26181, CIAT 26416 and CIAT 26570) were selected from a total of 66 accessions held in the gene bank of CIAT. Three *Brachiaria* grasses (*B. brizantha* cv. Toledo, *B. ruziziensis* 44-02 and a *B.* hybrid cv. Mulato II) with poorer adaptation to waterlogging than *B. humidicola* were included for reference (checks) but were excluded from analysis.

The soil used in this study was an Oxisol collected from Santander de Quilichao, Department of Cauca in Colombia (lat. 3°60′N; long. 76°310′W; altitude 990 m), 0–20 cm from the soil surface. All genotypes were grown from vegetative propagules and visually selected for homogeneity from 87-day-old plants growing in propagation pots. Each propagule had a single expanding leaf and a differentiated node for rooting **[see** **Supporting Information****]**. Propagules arose from stolons. Propagation pots consisted of plants (vegetative stage) that were growing in pots filled with 4 kg of a mixture of soil and sand (2 : 1 w/w) under pot capacity and fertilized conditions (milligrams added per kilogram of a soil–sand mixture: N 21, P 26, K 52, Ca 56, Mg 15, S 10, Zn 1.0, Cu 1.0, B 0.05 and Mo 0.05). Selected propagules were then washed for 1 min in 0.1 % commercial sodium hypochlorite before re-planting. A 1 : 1 (w/w) mixture of soil and sand was used to facilitate root growth and separation from soil for root analysis. Before re-planting of propagules, the soil mixture was thoroughly mixed with the following nutrient application (milligrams of element per kilogram of a soil–sand mixture: N 40, P 50, K 100, Ca 101, Mg 28, S 20, Zn 2.0, Cu 2.0, B 0.1 and Mo 0.1). This level of nutrient application represented the recommended fertility level for crop–pasture establishment (Rao *et al.* 1992). The soil mixture (4.5 kg) was packed in transparent plastic cylinders (80 cm high × 7.5 cm diameter) inserted into beige polyvinyl chloride (PVC) pipes. Three similarly sized propagules (∼6 cm length) were planted 2 cm below the soil surface in each soil cylinder, watered daily and thinned to one after 7 days. After thinning, propagules were watered daily and allowed to grow for another 21 days. A factorial combination of 15 genotypes by two drainage conditions (drained or waterlogged) was established in a four-replicate randomized complete block. Waterlogging treatment was imposed by sealing the lower end of the PVC pipes with a cap and maintaining a water level of 3 cm above the soil surface **[see** **Supporting Information****]**. Plants grown under drained soil were watered daily to maintain soil humidity at field capacity. The number of fully expanded leaves, leaf greenness and maximum rooting depth (cm) for each plant were recorded before the start of the experiment. Leaf greenness was measured in two fully expanded young leaves in SPAD (soil plant analysis development) units using a hand-held chlorophyll meter (SPAD-502, Konica Minolta, Japan). Maximum rooting depth was estimated from roots growing next to the wall of the transparent plastic cylinders.

The experiment was conducted in an open area at CIAT (Cali, Colombia). During the experiment, the average temperature was 31.5/23.0 °C (day/night), the relative air humidity was 41.2/56.8 % (day/night) and the maximum photosynthetic photon flux density was 1910 µmol m^−2^ s^−1^.

### Redox potentials

Redox potentials were monitored in four cylinders filled with a soil mixture (two for drained soil and two for waterlogged soil) using a platinum electrode and a calomel reference electrode connected to a micro-voltmeter (ODR meter, Eijkelkamp, The Netherlands) at three soil depths (∼5, 15 and 25 cm from the soil surface). Recording of measurements was taken after 3–5 min of equilibration before the start of the experiment (0 days) and after 21 days of treatment.

### Harvest

Before harvesting, the number of fully expanded leaves and maximum rooting depth (cm) for each plant were recorded. Maximum rooting depth was estimated from roots growing next to the wall of the transparent plastic cylinders that looked white and healthy. Plants were harvested after 21 days of growth under drained or waterlogged soil conditions. Previous work identified a period of ∼30 days of establishment plus 21 days of treatment as optimal to minimize limitations of root growth due to the container size of plants grown in soil cylinders of 80 cm height × 7.5 cm diameter. Shoot dry mass was determined after drying leaves and stems in an oven at 60 °C for 72 h. Each soil cylinder was sliced into four layers representing different depths from the soil surface (0–10, 10–20, 20–30 and 30–77 cm). To help remove rhizosphere soil from roots, each soil profile was placed in a container with a few drops of wetting agent (polysorbate 20) for 10–15 min and rinsed again with tap water to remove loosened soil. After washing, roots from each soil profile were stored separately in 50 % ethanol and stored at 4 °C for later analysis.

Using a dissecting microscope, the brighter and turgid live roots were easily distinguished from the darker and deflated dead ones, which were discarded. The number of live nodal roots was recorded. Thereafter, roots of each soil profile were placed in a tray filled with water and nodal and lateral roots were separated using a surgical scalpel blade. Lateral roots that appeared dead (blackish colour) together with organic matter debris were removed from the tray with an eyedropper. Images of nodal and lateral roots for each soil depth were recorded separately at 600 dpi using a flatbed dual scanner (EPSON Expression 1680, Japan). The length of nodal and lateral roots for each soil profile was estimated using the scanned images and WinRhizo software (Regent Instruments, Canada). After scanning, all roots were carefully collected to minimize loss of material, and oven dried at 60 °C for 72 h for the separate determination of dry mass of nodal and lateral roots down the soil profile. The overall proportion (%) of lateral roots (in terms of dry mass and length) to the total root system (sum of nodal and lateral roots) was determined. The proportions of lateral roots to the total root system for each soil profile were also calculated.

### Statistical analysis

Means, standard errors and analyses of variance (ANOVA) were calculated using the open source Agricolae Package of R (v. 2.15.2) (R Development Core Team 2012). Data were log transformed to ensure normality about the means. Differences between accessions were analysed using the least significant difference (LSD) at *α* = 0.05.

## Results

### Soil redox potentials

**Figure 1.**
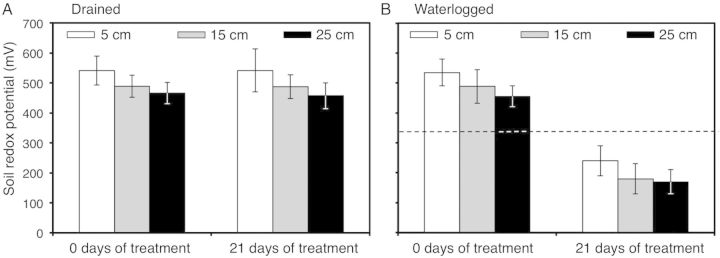
Soil redox potentials (mV) under (A) drained or (B) waterlogged conditions. Measurements were taken at ∼5, 15 and 25 cm from the soil surface. Columns represent means and error bars their standard error (*n* = 2). The dashed line represents the value where oxygen is undetectable (330 mV) (Ponnamperuma 1972).

Redox potentials maintained constant values of ∼500 mV under drained soil conditions (Fig. 1A) and decreased with waterlogging to values of ∼200 mV (Fig. 1B).

### Effect of waterlogging on leaf number, leaf greenness and dry mass production

**Figure 2.**
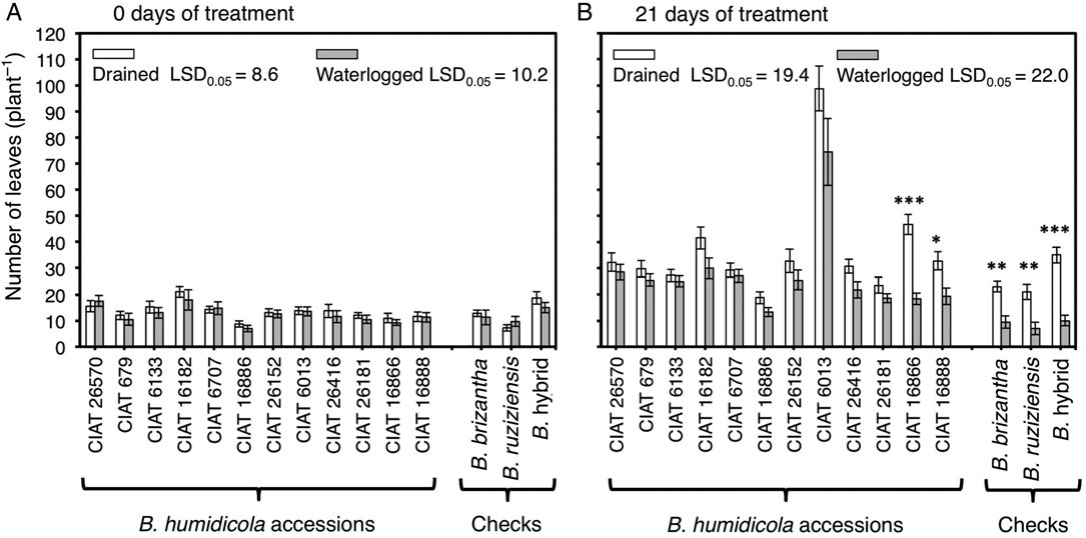
Number of leaves of 12 *B. humidicola* accessions (plus three checks): (A) before the start of the experiment and (B) after 21 days of growth under drained or waterlogged soil conditions. Columns represent means and error bars their standard error (*n* = 4). All accessions showed an increase in the number of leaves under both treatments from the beginning of the experiment (*P* < 0.05). Asterisks (*) represent significant differences between treatments for each accession (statistical significance at the *0.05, **0.01 and ***0.001 probability levels). Least significant difference values exclude checks.

**Figure 3.**
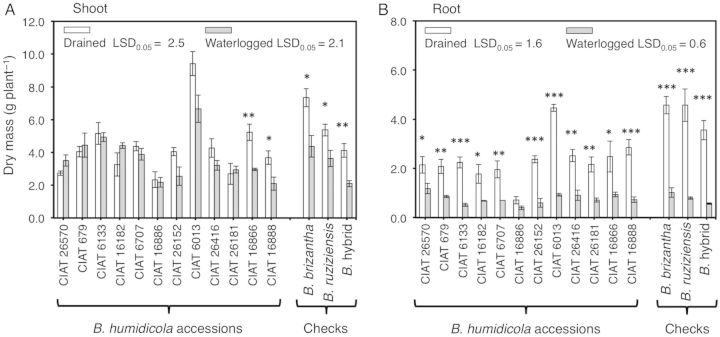
(A) Shoot and (B) root dry mass of 12 *B. humidicola* accessions (plus three checks) grown for 21 days under drained or waterlogged soil conditions. Columns represent means and error bars their standard error (*n* = 4). Asterisks (*) represent significant differences between treatments for each accession (statistical significance at the *0.05, **0.01 and ***0.001 probability levels). Least significant difference values exclude checks.

From the beginning of the experiment, leaf production continued irrespective of treatment (Fig. 2A and B). Two accessions (CIAT 16866 and CIAT 16888) showed a reduction in the number of leaves when grown under waterlogging (*P* < 0.05; Fig. 2B). Waterlogging did not promote leaf senescence in any of the accessions tested (Table 1). Waterlogging significantly reduced shoot dry mass in CIAT 16888 and CIAT 16866 (Fig. 3A) and root dry mass for all accessions except CIAT 16886 (Fig. 3B).

**Table 1.** Leaf greenness (SPAD units) and dead leaf dry mass of 12 *B. humidicola* accessions (plus three checks) grown under drained or waterlogged soil. Data shown are means of four replicates ± SE. An asterisk (*) represents significant differences between treatments for each accession (statistical significance at the *0.05, **0.01 and ***0.001 probability levels). *P*_anova_ and LSD values exclude checks.

Accession	Leaf greenness (SPAD units)				Dead leaf dry mass (g plant^−1^)	
	0 days of treatment		21 days of treatment		21 days of treatment	
	Drained	Waterlogged	Drained	Waterlogged	Drained	Waterlogged
CIAT 26570	36.4 ± 1.1	34.8 ± 1.4	32.4 ± 1.9	30.1 ± 1.9	0.22 ± 0.06	0.33 ± 0.03
CIAT 679	40.9 ± 1.2	38.4 ± 2.8	35.6 ± 2.7	33.9 ± 1.5	0.26 ± 0.06	0.36 ± 0.04
CIAT 6133	38.4 ± 2.0	39.6 ± 2.8	35.7 ± 1.3	34.5 ± 2.9	0.12 ± 0.01	0.11 ± 0.04
CIAT 16182	43.0 ± 2.2	39.5 ± 2.4	38.0 ± 1.6	33.4 ± 1.7	0.10 ± 0.02	0.18 ± 0.02
CIAT 6707	38.1 ± 1.4	38.3 ± 1.5	35.8 ± 1.1	33.3 ± 1.6	0.09 ± 0.03	0.19 ± 0.03
CIAT 16886	37.1 ± 2.2	38.2 ± 1.3	36.0 ± 1.5	35.5 ± 1.6	0.10 ± 0.02	0.11 ± 0.02
CIAT 26152	43.5 ± 2.9	41.0 ± 2.3	38.5 ± 2.9	38.6 ± 1.1	0.46 ± 0.13	0.31 ± 0.09
CIAT 6013	35.5 ± 2.2	35.6 ± 2.7	29.2 ± 2.6	28.6 ± 2.0	0.45 ± 0.10	0.41 ± 0.11
CIAT 26416	37.0 ± 2.1	36.2 ± 2.6	34.0 ± 1.3	30.1 ± 2.8	0.04 ± 0.05	0.10 ± 0.03
CIAT 26181	41.0 ± 2.9	39.6 ± 2.4	34.7 ± 2.1	30.5 ± 3.1	0.11 ± 0.03	0.22 ± 0.09
CIAT 16866	41.6 ± 3.1	40.3 ± 1.8	37.6 ± 2.0	36.3 ± 2.0	0.11 ± 0.04	0.21 ± 0.02
CIAT 16888	39.7 ± 3.7	38.4 ± 1.5	38.9 ± 1.8	33.7 ± 2.1	0.05 ± 0.01	0.09 ± 0.02
*P*_anova_	0.3016	0.6732	0.0702	0.0664	0.0000	0.0002
LSD_0.05_	NS	NS	NS	NS	0.27	0.24
Checks						
*B. brizantha*	36.4 ± 1.4	39.9 ± 1.6	29.5 ± 3.0	20.8 ± 1.5***	0.15 ± 0.04	0.56 ± 0.07**
*B. ruziziensis*	45.9 ± 2.5	47.3 ± 1.9	31.5 ± 2.6	12.7 ± 1.9***	0.33 ± 0.06	1.22 ± 0.10***
*B*. hybrid	39.4 ± 1.4	43.9 ± 1.1	32.7 ± 2.7	18.1 ± 1.4***	0.08 ± 0.01	0.44 ± 0.07**

### Effect of waterlogging on root architecture

**Figure 4.**
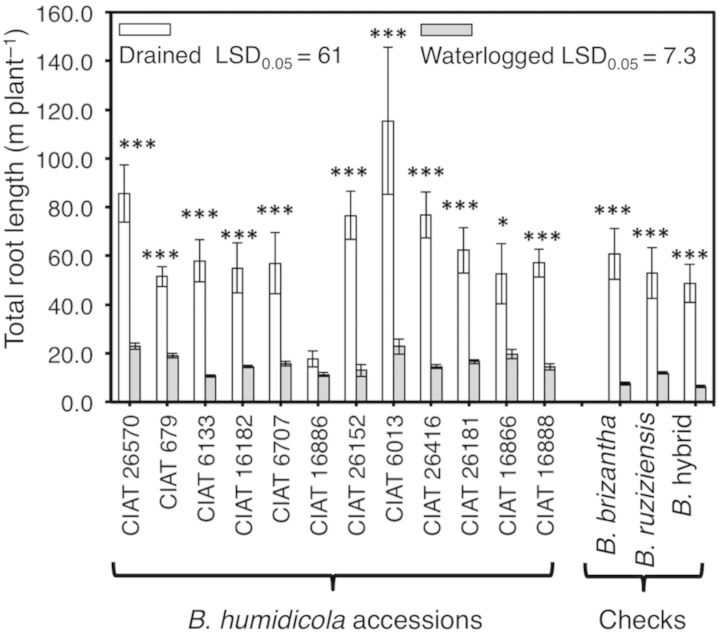
Total root length (m plant^−1^) of 12 *B. humidicola* accessions (plus three checks) grown for 21 days under drained or waterlogged soil conditions. Columns represent means and error bars their standard error (*n* = 4). Asterisks (*) represent significant differences between treatments for each accession (statistical significance at the ***0.001 probability levels). Least significant difference values exclude checks.

In the present study, there was a tendency for an increase in the number of nodal roots per plant under waterlogged conditions, but this was not statistically significant for any of the tested accessions (Table 2). Under waterlogging conditions, the number of nodal roots for each soil profile was reduced with increasing depth **[see** **Supporting Information****]**. Before the start of the experiment, the maximum rooting depth of all accessions was within the range of 15–25 cm below the soil surface (Table 2). Under drained soil conditions, nodal roots of all *B. humidicola* accessions reached the bottom of the cylinders (77 cm depth) before the end of the experiment. Conversely, nodal roots of *B. humidicola* accessions did not grow deeper than 30 cm into waterlogged soil (Table 2). Total root length was significantly reduced by waterlogging in all accessions except for CIAT 16886 (Fig. 4).

**Table 2.** Number of nodal roots and maximum rooting depth of 12 *B. humidicola* accessions (plus three checks) grown under drained or waterlogged soil. Nodal roots of plants grown under drained soil reached the bottom of the cylinders (77 cm). Data shown are means of four replicates ± SE. NS, not significant. *P*_anova_ and LSD values exclude checks.

Accession	Number of nodal roots (plant^−1^)		Maximum rooting depth (cm plant^−1^)		
	21 days of treatment		0 days of treatment		21 days of treatment
	Drained	Waterlogged	Drained	Waterlogged	Waterlogged
CIAT 26570	27.0 ± 0.6	30.3 ± 2.3	19.3 ± 2.2	18.8 ± 2.6	23.0 ± 2.0
CIAT 679	22.3 ± 1.8	29.3 ± 2.3	19.8 ± 3.7	18.8 ± 2.7	22.5 ± 1.3
CIAT 6133	18.8 ± 1.9	26.3 ± 3.3	23.0 ± 3.7	22.8 ± 2.3	22.8 ± 1.7
CIAT 16182	23.8 ± 2.9	25.0 ± 4.1	23.0 ± 3.7	22.3 ± 3.2	17.8 ± 1.4
CIAT 6707	20.3 ± 3.2	20.0 ± 2.8	21.3 ± 3.4	22.3 ± 2.2	21.3 ± 2.1
CIAT 16886	20.3 ± 2.4	30.8 ± 3.1	15.8 ± 1.9	17.5 ± 1.7	19.0 ± 1.8
CIAT 26152	28.5 ± 6.5	35.0 ± 5.9	22.8 ± 3.2	17.5 ± 1.7	21.8 ± 1.3
CIAT 6013	28.3 ± 2.5	35.0 ± 3.4	24.5 ± 2.5	25.5 ± 2.6	22.5 ± 2.7
CIAT 26416	34.0 ± 3.6	36.3 ± 3.3	21.3 ± 3.1	20.0 ± 2.7	20.9 ± 1.9
CIAT 26181	18.8 ± 2.5	28.8 ± 3.3	21.5 ± 2.3	23.0 ± 2.8	19.0 ± 0.9
CIAT 16866	29.5 ± 2.4	34.0 ± 3.1	18.3 ± 3.4	19.5 ± 2.4	21.8 ± 2.1
CIAT 16888	32.8 ± 4.1	39.3 ± 4.9	19.5 ± 2.4	17.5 ± 1.7	19.5 ± 1.9
*P*_anova_	0.0083	0.0464	0.7722	0.2570	0.5767
LSD_0.05_	15.3	18.1	NS	NS	NS
Checks					
*B. brizantha*	29.5 ± 3.3	25.8 ± 2.8	33.8 ± 3.5	31.5 ± 4.1	12.0 ± 0.6
*B. ruziziensis*	40.0 ± 5.6	39.5 ± 6.9	32.5 ± 4.1	31.8 ± 4.7	10.8 ± 1.0
*B.* hybrid	32.0 ± 6.2	13.8 ± 1.9	18.0 ± 2.7	18.5 ± 2.3	9.6 ± 0.9

**Figure 5.**
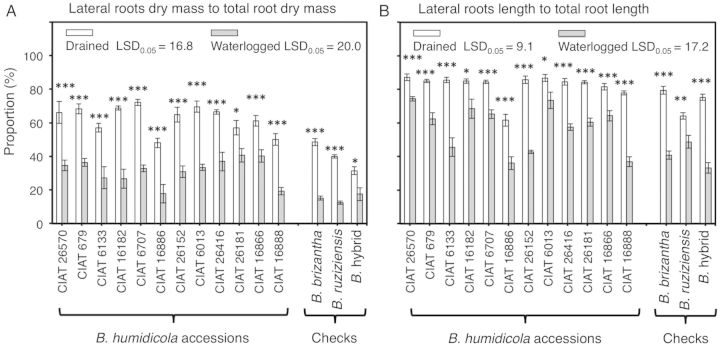
Overall proportion of (A) lateral root dry mass to total root weight and (B) lateral root length to total root length of 12 *B. humidicola* accessions (plus three checks) grown for 21 days under drained or waterlogged soil conditions. Columns represent means and error bars their standard error (*n* = 4). Asterisks (*) represent significant differences between treatments for each accession (statistical significance at the *0.05, **0.01 and ***0.001 probability levels). Least significant difference values exclude checks.

**Figure 6.**
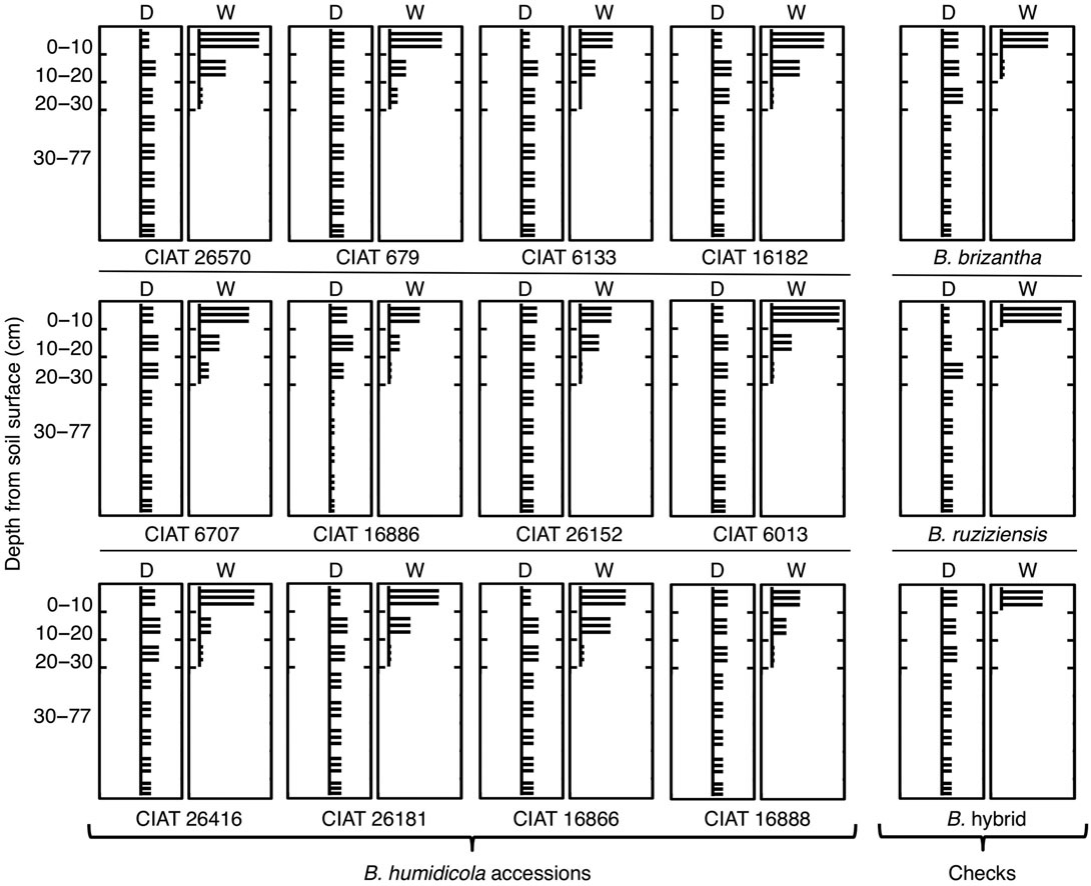
Schematic representation of the relative contribution of lateral root length across soil depth in 12 *B. humidicola* accessions (plus three checks) grown for 21 days under drained or waterlogged soil conditions. Horizontal lines represent lateral roots. Vertical lines represent nodal roots. Distribution of lateral roots at 30–77 cm of soil depth under drained soil conditions was assumed to be even.

Waterlogging changed root dry mass and length down the soil profile for all accessions (Tables 3 and 4). Root dry mass and root length progressively decreased from the upper layer of the soil to increasing depth under waterlogged soil conditions (Tables 3 and 4). When grown under drained soil conditions, the lateral root system of all *B. humidicola* accessions comprised at least 50 % of the total of root dry mass and over 60 % of the length of the total root system (Fig. 5A and B). Waterlogging decreased the proportion of lateral roots to the entire root system in terms of both dry mass and length (Fig. 5A and B). Waterlogging also induced changes in the proportion of lateral roots (dry mass and length) at different depths in the soil in all accessions (Tables 5 and 6; Fig. 6). In drained soil, the proportion of lateral roots of all accessions was relatively evenly distributed in the top 30 cm of soil depth (Tables 5 and 6; Fig. 6). Conversely, waterlogging increased the proportion of lateral roots to the upper layers of the soil (Tables 5 and 6; Fig. 6). Under waterlogged soil and in all accessions, there was a tendency for lateral roots in the top 10 cm of soil to grow towards the soil surface (visual observation of negative gravitropism).

**Table 3.** Root dry mass distribution across soil depth of 12 *B. humidicola* accessions (plus three checks) grown under drained or waterlogged soil for 21 days. Data shown are means of four replicates ± SE. Asterisks (*) represent significant differences between treatments for each accession (statistical significance at the *0.05, **0.01 and ***0.001 probability levels). NS, not significant. *P*_anova_ and LSD values exclude checks.

Accession	Root dry mass (g plant^−1^)						
	0–10 cm		10–20 cm		20–30 cm		30–77 cm
	Drained	Waterlogged	Drained	Waterlogged	Drained	Waterlogged	Drained
CIAT 26570	0.37 ± 0.02	0.88 ± 0.19*	0.43 ± 0.05	0.22 ± 0.00**	0.31 ± 0.02	0.08 ± 0.01***	1.04 ± 0.29
CIAT 679	0.32 ± 0.06	0.55 ± 0.03*	0.28 ± 0.04	0.20 ± 0.02	0.20 ± 0.03	0.11 ± 0.02	1.28 ± 0.23
CIAT 6133	0.43 ± 0.05	0.33 ± 0.03	0.36 ± 0.06	0.11 ± 0.02**	0.28 ± 0.03	0.08 ± 0.02**	1.16 ± 0.13
CIAT 16182	0.32 ± 0.07	0.45 ± 0.04	0.31 ± 0.07	0.15 ± 0.03	0.26 ± 0.06	0.08 ± 0.02*	0.90 ± 0.20
CIAT 6707	0.27 ± 0.03	0.47 ± 0.02**	0.27 ± 0.05	0.14 ± 0.03	0.27 ± 0.07	0.09 ± 0.03	1.15 ± 0.20
CIAT 16886	0.24 ± 0.06	0.31 ± 0.06	0.15 ± 0.02	0.07 ± 0.02*	0.11 ± 0.02	0.02 ± 0.01*	0.21 ± 0.05
CIAT 26152	0.65 ± 0.08	0.38 ± 0.09	0.34 ± 0.03	0.13 ± 0.05*	0.31 ± 0.10	0.09 ± 0.04	1.06 ± 0.09
CIAT 6013	0.78 ± 0.19	0.62 ± 0.05	0.54 ± 0.11	0.21 ± 0.02*	0.57 ± 0.19	0.10 ± 0.02	2.56 ± 0.88
CIAT 26416	0.59 ± 0.05	0.52 ± 0.08	0.49 ± 0.02	0.26 ± 0.08*	0.38 ± 0.04	0.14 ± 0.05**	1.06 ± 0.14
CIAT 26181	0.41 ± 0.04	0.45 ± 0.05	0.34 ± 0.05	0.17 ± 0.02*	0.34 ± 0.04	0.09 ± 0.03**	1.07 ± 0.21
CIAT 16866	0.43 ± 0.09	0.50 ± 0.03	0.35 ± 0.08	0.28 ± 0.06	0.37 ± 0.08	0.17 ± 0.05	1.32 ± 0.39
CIAT 16888	0.72 ± 0.06	0.47 ± 0.06	0.60 ± 0.10	0.18 ± 0.03**	0.39 ± 0.05	0.07 ± 0.02***	1.15 ± 0.14
*P*_anova_	0.0002	0.0008	0.0011	0.0165	0.0369	0.1330	0.0146
LSD_0.05_	0.4	0.4	0.3	0.2	0.4	–	1.6
Checks							
*B. brizantha*	1.17 ± 0.09	0.86 ± 0.16	0.96 ± 0.07	0.16 ± 0.04***	0.75 ± 0.09	–	1.69 ± 0.29
*B. ruziziensis*	0.94 ± 0.07	0.79 ± 0.05	0.67 ± 0.09	–	0.62 ± 0.11	–	2.34 ± 0.39
*B*. hybrid	1.68 ± 0.22	0.57 ± 0.03***	0.57 ± 0.12	–	0.57 ± 0.11	–	0.74 ± 0.19

**Table 4.** Root length distribution across soil depth of 12 *B. humidicola* accessions (plus three checks) grown under drained or waterlogged soil for 21 days. Data shown are means of four replicates ± SE. Asterisks (*) represent significant differences between treatments for each accession (statistical significance at the *0.05, **0.01 and ***0.001 probability levels). NS, not significant. *P*_anova_ and LSD values exclude checks.

Accession	Root length (m plant^−1^)						
	0–10 cm		10–20 cm		20–30 cm		30–77 cm
	Drained	Waterlogged	Drained	Waterlogged	Drained	Waterlogged	Drained
CIAT 26570	8.4 ± 0.7	14.4 ± 0.1***	13.1 ± 1.1	7.0 ± 1.0**	10.4 ± 0.8	1.6 ± 0.2***	53.6 ± 9.2
CIAT 679	7.6 ± 0.4	12.4 ± 0.5***	6.5 ± 0.8	4.2 ± 0.2*	6.8 ± 1.5	2.4 ± 0.2*	30.5 ± 1.5
CIAT 6133	6.0 ± 1.2	6.5 ± 0.3	9.6 ± 1.6	3.1 ± 0.2**	7.5 ± 0.5	1.2 ± 0.1***	34.9 ± 5.4
CIAT 16182	6.8 ± 1.0	9.7 ± 0.3*	10.0 ± 1.7	4.1 ± 0.2*	9.0 ± 2.0	0.8 ± 0.0**	29.3 ± 5.5
CIAT 6707	7.8 ± 0.5	9.8 ± 0.5*	9.6 ± 1.8	4.3 ± 0.4*	9.4 ± 2.6	1.8 ± 0.2*	30.1 ± 7.6
CIAT 16886	5.0 ± 0.9	7.3 ± 0.2*	4.7 ± 0.4	2.7 ± 0.4*	3.5 ± 0.6	1.2 ± 0.2*	4.5 ± 1.4
CIAT 26152	14.6 ± 2.8	8.1 ± 1.5	11.5 ± 3.6	3.3 ± 0.7	10.4 ± 3.2	1.6 ± 0.3*	40.1 ± 0.2
CIAT 6013	14.9 ± 3.1	16.8 ± 2.3	16.6 ± 2.9	5.0 ± 0.7**	16.8 ± 5.0	1.0 ± 0.1*	67.1 ± 19.8
CIAT 26416	13.6 ± 0.8	10.6 ± 0.2**	14.7 ± 1.3	2.6 ± 0.3***	12.9 ± 1.6	1.2 ± 0.3***	35.5 ± 5.7
CIAT 26181	8.5 ± 1.3	10.9 ± 0.3	10.7 ± 1.3	4.4 ± 0.5**	9.5 ± 2.0	1.3 ± 0.1**	33.6 ± 4.8
CIAT 16866	6.9 ± 1.0	11.4 ± 0.5**	7.5 ± 1.4	6.7 ± 1.1	8.3 ± 1.9	1.5 ± 0.2*	30.0 ± 8.1
CIAT 16888	12.3 ± 1.1	9.5 ± 0.7	9.2 ± 2.0	3.8 ± 0.5**	8.3 ± 1.3	1.2 ± 0.1**	27.3 ± 1.4
*P*_anova_	0.0000	0.0000	0.0031	0.0000	0.0491	0.0004	0.0012
LSD_0.05_	7.1	4.2	8.9	2.9	10.9	1.0	37.3
Checks							
*B. brizantha*	11.4 ± 0.7	6.7 ± 0.6**	11.0 ± 2.2	1.0 ± 0.1***	11.9 ± 1.4	–	26.5 ± 6.2
*B. ruziziensis*	8.1 ± 0.5	11.9 ± 0.4***	7.7 ± 1.1	–	7.2 ± 1.7		30.0 ± 7.2
*B*. hybrid	11.0 ± 0.6	6.3 ± 0.4****	7.7 ± 1.2	–	8.3 ± 1.3		21.6 ± 4.6

**Table 5.** Proportion (%) of lateral root dry mass to total root dry mass across soil depth of 12 *B. humidicola* accessions (plus three checks) grown under drained or waterlogged soil for 21 days. Data shown are means of four replicates ± SE. Asterisks (*) represent significant differences between treatments for each accession (statistical significance at the *0.05, **0.01 and ***0.001 probability levels). NS, not significant. *P*_anova_ and LSD values exclude checks.

Accession	Proportion (%) of lateral root dry mass by soil profile						
	0–10 cm		10–20 cm		20–30 cm		30–77 cm
	Drained	Waterlogged	Drained	Waterlogged	Drained	Waterlogged	Drained
CIAT 26570	9.2 ± 2.4	22.7 ± 2.7**	12.3 ± 0.9	9.4 ± 0.8	10.3 ± 1.9	2.5 ± 0.5*	34.3 ± 11.6
CIAT 679	6.1 ± 1.0	26.3 ± 1.3***	6.1 ± 0.2	6.6 ± 1.3	7.0 ± 1.0	3.5 ± 0.6*	49.1 ± 2.8
CIAT 6133	5.1 ± 1.0	14.3 ± 4.6	9.2 ± 1.2	8.0 ± 1.7	6.9 ± 0.7	4.9 ± 0.3*	35.8 ± 3.5
CIAT 16182	6.4 ± 1.0	16.2 ± 3.2*	11.1 ± 1.3	7.4 ± 1.8	10.8 ± 0.5	3.1 ± 0.9***	40.5 ± 0.5
CIAT 6707	6.3 ± 0.5	21.1 ± 1.7***	9.8 ± 0.5	8.0 ± 2.2	10.3 ± 1.3	3.8 ± 1.4*	45.8 ± 3.0
CIAT 16886	10.3 ± 2.4	13.9 ± 4.5	13.5 ± 2.8	2.7 ± 0.9**	9.0 ± 0.3	1.1 ± 0.4***	15.3 ± 2.6
CIAT 26152	9.4 ± 1.1	18.9 ± 2.8*	9.1 ± 2.2	7.8 ± 0.5	9.0 ± 3.3	4.2 ± 0.4	37.3 ± 3.2
CIAT 6013	7.4 ± 1.5	22.0 ± 1.3***	8.4 ± 1.4	7.7 ± 1.9	9.1 ± 0.7	3.7 ± 1.2**	44.6 ± 3.5
CIAT 26416	9.8 ± 1.3	16.9 ± 1.5**	12.8 ± 1.0	14.7 ± 4.2	11.6 ± 0.9	5.3 ± 2.2*	32.2 ± 3.0
CIAT 26181	4.6 ± 2.2	26.7 ± 2.7**	8.9 ± 0.9	10.8 ± 1.1	10.7 ± 0.6	3.2 ± 0.3***	32.9 ± 5.5
CIAT 16866	5.7 ± 1.0	20.9 ± 1.6***	8.3 ± 1.5	13.2 ± 3.3	11.6 ± 2.8	6.2 ± 2.0	35.6 ± 3.6
CIAT 16888	7.2 ± 0.6	10.7 ± 1.1*	7.6 ± 1.4	6.4 ± 0.8	7.7 ± 1.0	2.2 ± 0.4**	27.6 ± 1.6
*P*_anova_	0.0983	0.0023	0.0028	0.0181	0.3280	0.1204	0.0009
LSD_0.05_	–	12.9	6.9	9.7	–	–	21.9
Checks							
*B. brizantha*	5.7 ± 0.2	10.6 ± 1.1**	9.7 ± 0.8	4.5 ± 1.3*	8.8 ± 0.3	–	24.4 ± 2.5
*B. ruziziensis*	1.9 ± 0.1	12.4 ± 1.0***	3.9 ± 0.0	–	5.1 ± 0.5	–	29.0 ± 0.6
*B*. hybrid	7.6 ± 0.2	17.5 ± 3.9*	5.5 ± 0.5	–	6.1 ± 0.9	–	12.3 ± 3.2

**Table 6.** Proportion (%) of lateral root length to total root length across soil depth of 12 *B. humidicola* accessions (plus three checks) grown under drained or waterlogged soil for 21 days. Data shown are means of four replicates ± SE. Asterisks (*) represent significant differences between treatments for each accession (statistical significance at the *0.05, **0.01 and ***0.001 probability levels). NS, not significant. *P*_anova_ and LSD values exclude checks.

Accession	Proportion (%) of lateral root length by soil profile						
	0–10 cm		10–20 cm		20–30 cm		30–77 cm
	Drained	Waterlogged	Drained	Waterlogged	Drained	Waterlogged	Drained
CIAT 26570	7.1 ± 0.5	49.5 ± 3.3***	12.6 ± 1.2	22.0 ± 3.3*	10.1 ± 0.9	3.0 ± 0.4***	57.3 ± 3.9
CIAT 679	11.0 ± 0.6	42.7 ± 2.9***	9.2 ± 0.6	13.3 ± 1.2*	10.9 ± 1.8	6.5 ± 1.0	54.0 ± 1.2
CIAT 6133	6.7 ± 1.9	26.7 ± 3.9**	13.4 ± 2.0	12.4 ± 0.9	11.0 ± 0.8	6.5 ± 0.9**	54.5 ± 4.6
CIAT 16182	7.7 ± 0.7	43.3 ± 4.1***	15.5 ± 2.0	23.2 ± 2.4*	13.8 ± 1.4	2.0 ± 0.1***	48.0 ± 1.7
CIAT 6707	10.2 ± 1.4	40.9 ± 2.0***	14.4 ± 1.3	16.5 ± 0.7	14.3 ± 1.0	7.8 ± 1.0***	45.6 ± 2.5
CIAT 16886	13.9 ± 1.4	25.2 ± 3.0*	18.8 ± 1.4	8.7 ± 1.9**	11.0 ± 1.3	2.1 ± 0.2***	18.0 ± 4.1
CIAT 26152	12.9 ± 1.5	25.6 ± 0.1***	11.3 ± 2.6	15.4 ± 0.9	10.8 ± 2.3	1.8 ± 0.1**	50.7 ± 5.2
CIAT 6013	8.7 ± 1.1	55.2 ± 4.3***	12.8 ± 1.6	16.3 ± 1.6	12.5 ± 0.8	2.1 ± 0.0***	52.8 ± 3.0
CIAT 26416	11.8 ± 0.9	45.0 ± 2.8***	16.0 ± 1.1	9.5 ± 0.5**	14.9 ± 1.2	3.0 ± 0.8***	41.8 ± 4.3
CIAT 26181	8.8 ± 0.6	40.8 ± 1.3***	14.7 ± 2.9	17.7 ± 1.8	12.6 ± 1.4	2.1 ± 0.5**	48.1 ± 1.5
CIAT 16866	7.5 ± 0.4	37.0 ± 3.4***	11.5 ± 1.3	24.3 ± 4.8*	13.9 ± 2.7	3.0 ± 0.5**	48.7 ± 5.7
CIAT 16888	12.2 ± 1.4	23.0 ± 2.5**	11.7 ± 1.4	11.8 ± 1.2	12.0 ± 1.1	2.1 ± ***	41.9 ± 1.1
*P*_anova_	0.0001	0.0000	0.0375	0.0000	0.3646	0.0000	0.0000
LSD_0.05_	5.4	14.5	8.4	10.2	–	2.8	16.7
Checks							
*B. brizantha*	13.0 ± 1.7	38.2 ± 2.5**	13.7 ± 1.5	2.6 ± 0.4***	16.8 ± 0.9	–	35.9 ± 4.2
*B. ruziziensis*	5.8 ± 2.8	48.7 ± 3.8**	7.7 ± 1.2	–	7.8 ± 1.0	–	42.9 ± 4.2
*B*. hybrid	12.3 ± 1.7	33.3 ± 3.2*	11.4 ± 1.0	–	12.3 ± 1.0	–	39.3 ± 3.4

## Discussion

Waterlogging for 21 days reduced the shoot dry mass of accessions CIAT 16866 and CIAT 16888 together with a decrease in the number of leaves. This reflects the importance of leaf production in maintaining shoot growth (Figs 2 and 3). In the present study, reduction of leaf number in CIAT 16866 and CIAT 16888 when waterlogged was not the outcome of faster leaf senescence (Table 1), but rather a result of slower leaf production (Fig. 2). Total root dry mass and total root length were both significantly reduced in *B. humidicola* accessions (except CIAT 16886) under waterlogging conditions (Figs 3B and 4). In contrast, total root dry mass and total root length of CIAT 16886 were not reduced, possibly a reflection of its inherently slower growth rate as indicated by the lower production of leaves throughout the period of study (Fig. 2A and B). For any accession, there was no reduction in the number of nodal roots per plant under waterlogging conditions. Thus, the decreases in total root dry mass and total root length are attributable to a restriction of nodal root penetration below 30 cm in anoxic soil (Figs 3 and 4; Table 2). The maximum rooting depth into waterlogged and anoxic soils depends on the amount of oxygen that reaches the root tip (Colmer 2003; Colmer and Greenway 2005). This is greatly facilitated by aerenchyma (Justin and Armstrong 1987; Armstrong and Drew 2002; Colmer 2003; Colmer and Greenway 2005). Previous research showed that nodal roots of *B. humidicola* responded to waterlogging by increasing root aerenchyma (to around 30 % of cross-sectional area); this is presumably to improve root aeration and sustain elongation (Cardoso *et al.* 2013).

Our data showed that waterlogged soil reached anoxic conditions (<330 mV; Fig. 1). Although the importance of aerenchyma in supporting root elongation under oxygen-deficient conditions is widely recognized, it was shown that even roots with very high porosities (over 30 %) rarely penetrate more than 30 cm of soil (e.g. wetland species; Justin and Armstrong 1987). On this basis, roots of *B. humidicola* would also be expected to be limited to 30 cm in flooded soil. This in turn will restrict total root surface area to one that restricts nutrient and water uptake to maintain shoot growth. Therefore, we suggest that other adaptations acting together with aerenchyma development sustain the shoot growth of *B. humidicola* under waterlogging conditions.

The present study shows that lateral roots comprise a large component of the root system. In all the *B. humidicola* accessions we tested, lateral roots represented around 50 % of total root weight and 62–88 % of total root length under drained conditions (Fig. 5A and B). Under waterlogging conditions, an overall reduction of the proportion of lateral roots (in terms of both dry mass and length) to total root length was found (Fig. 5A and B). A reduction of lateral roots under oxygen shortage has previously been recorded for some accessions of *B. humidicola* (Cardoso *et al.* 2013) and other species such as rice (Ota 1970), pea (Armstrong *et al.* 1983), sorghum (Pardales *et al.* 1991) and banana (Aguilar *et al.* 2003). It has been argued that a reduction of lateral roots developed from the parent root is of adaptive value as more oxygen could reach the tip of the parent root to sustain root elongation (Armstrong *et al.* 1983; Sorrell *et al.* 2000; Aguilar *et al.* 2003). However, this will probably result in a reduction of root surface area, already restricted by the maximum penetration attained by roots into waterlogged soil.

The present study showed that there was a significant increase in the proportion of lateral root length in the upper 10 cm of waterlogged soil (Table 6; Fig. 6). An increase in the proportion of lateral roots in the top 10 cm of soil suggests an enhanced development of lateral roots closer to the root base of nodals under waterlogging conditions. Increased lateral root development closer to the root base might be a consequence of slower nodal root elongation under waterlogging conditions. Another possibility is that lateral root development near the root base was facilitated by a closer proximity to the oxygen provided by aerenchyma from the nodal root. According to Armstrong *et al.* (1990), O_2_ consumed by lateral roots developed from the base of parent roots with high porosity will have little impact on the O_2_ concentration in the root base and thus in the O_2_ diffusion path to the root tip. Therefore, it is possible that the bulk of lateral roots of *B. humidicola* accessions were initiated within the first 10 cm of the waterlogged soil. This could be considered advantageous under waterlogged conditions since they would not detrimentally affect the penetration of the parent root (i.e. nodal roots with aerenchyma).

Although the focus of this study was on *B. humidicola* accessions, it is instructive to note the responses of *B. brizantha*, *B. ruziziensis* and a *Brachiaria* hybrid that were used to confirm the superior waterlogging tolerance of the selected *B. humidicola* accessions. Their relatively poor adaptation when compared with *B. humidicola* was revealed as a more strongly reduced shoot growth and increased leaf senescence (Table 1; Fig. 3). This greater damage above ground was associated with confinement of the roots to the top 10 cm of waterlogged soil (the maximum rooting depth was ∼2-fold below that of the *B. humidicola* accessions; Table 2). Shallower root systems were presumably a consequence of less aerenchyma in roots of the three check species since this has previously been found to be approximately half that in *B. humidicola* accessions and comprise ∼16 % of cross-sectional area (Cardoso *et al.* 2013).

## Conclusions

The present study shows that the overall proportion of lateral roots developed from the parent root axes of 12 accessions of *B. humidicola* was decreased by 21 days of soil waterlogging. However, in the top 10 cm of the soil the reverse was the case, with waterlogging increasing the proportion of the roots that comprised lateral roots. An increased proportion of lateral roots in the upper layers is thought to be of adaptive value by compensating for the reduction of absorptive root surface resulting from the inhibition of root growth at depths below 30 cm.

Variation among the accessions was found for all plant attributes and proportions measured. However, it was not possible to establish associations between root traits and the reduction of shoot growth under waterlogging conditions for two accessions (CIAT 16866 and CIAT 16888). Further research is therefore needed to establish the differences in the efficiency of nutrient and water uptake by both nodal (aerenchymatous) and lateral (non-aerenchymatous) roots and test their relationships with the observed differences in shoot growth among *B. humidicola* accessions. A causal connection between lack of rooting depth and extent of aerenchyma is indicated by the shallow rooting depth of waterlogging-intolerant *Brachiaria* species that characteristically have much less extensive aerenchyma in their main root axes.

## Sources of Funding

This work was partially funded by FONTAGRO (USA) (project number: FTG-8060/08).

## Contributions by the Authors

J.A.C. was involved in designing the experiments, data collection and analysis, manuscript preparation and submission. J.C.J. contributed to the set-up of experiments and manuscript preparation. I.M.R. was involved in designing and supervision of experiments, manuscript preparation and submission.

## Conflicts of Interest Statement

None declared.

## Supplementary Material

Additional Information

## Supporting Information

The following Supporting Information is available in the online version of this article –

**File 1.** Schematic representation of system used to evaluate waterlogging tolerance in *Brachiaria* spp.

**File 2.** Number of nodal roots across soil depth in 12 *B. humidicola* accessions (plus three checks) grown under drained or waterlogged soil for 21 days. Data shown are means of four replicates ± SE. Number of nodal roots for each soil profile was reduced with increasing depth.
