# Supplementary material for: Waterlogging-induced changes in root architecture of germplasm accessions of the tropical forage grass Brachiaria humidicola
Source: AoB Plants. 2014 Apr 8;6:plu017. doi: 10.1093/aobpla/plu017 (PMC4038435; doi:10.1093/aobpla/plu017)
Supplement: Additional Information [file supp_6_plu017_index.html]

Waterlogging-induced changes in root architecture of germplasm accessions of the tropical forage grass Brachiaria humidicola — Additional Information 

# Waterlogging-induced changes in root architecture of germplasm accessions of the tropical forage grass *Brachiaria humidicola*

## Additional Information

Additional Information

**Files in this Data Supplement:**

- Additional Information - docx file
- Additional Information - docx file
