# Supplementary material for: Waterlogging-induced changes in root architecture of germplasm accessions of the tropical forage grass Brachiaria humidicola
Source: AoB Plants. 2014 Apr 8;6:plu017. doi: 10.1093/aobpla/plu017 (PMC4038435; doi:10.1093/aobpla/plu017)
Supplement: Additional Information [file supp_plu017_plu017supp_fig1.doc]

**SUPPORTING INFORMATION**

**File 1. Figure. Schematic representation of system used to evaluate waterlogging tolerance in *Brachiaria* spp.**

**
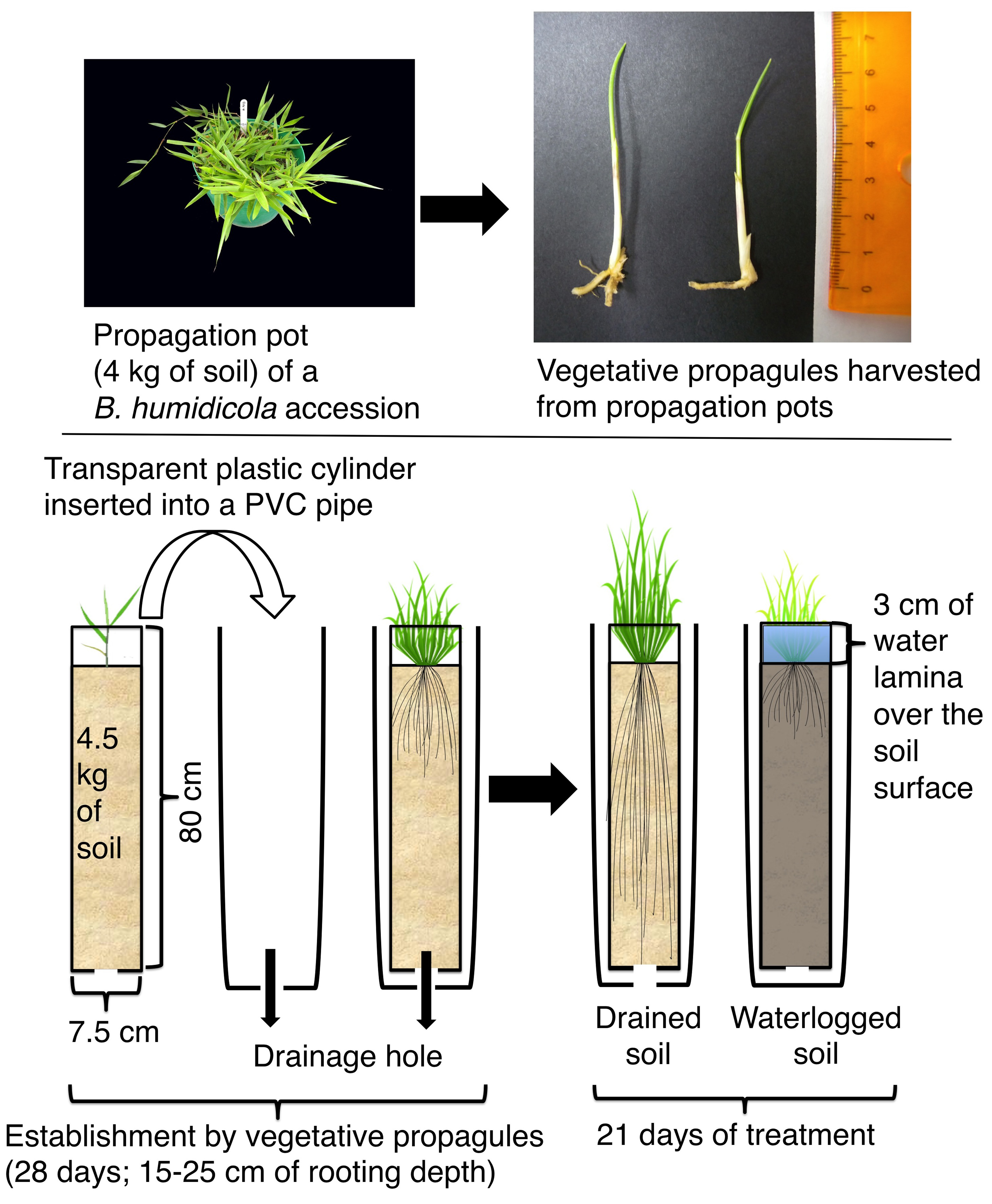
**
