# Supplementary material for: Waterlogging-induced changes in root architecture of germplasm accessions of the tropical forage grass Brachiaria humidicola
Source: AoB Plants. 2014 Apr 8;6:plu017. doi: 10.1093/aobpla/plu017 (PMC4038435; doi:10.1093/aobpla/plu017)

**SUPPORTING INFORMATION**

**File 2. Figure. Number of nodal roots across soil depth of 12 *B. humidicola* accessions (plus three checks) grown under drained or waterlogged soil for 21 days.** Data shown are means of four replicates ± S.E. Number of nodal roots for each soil profile was reduced with increasing depth


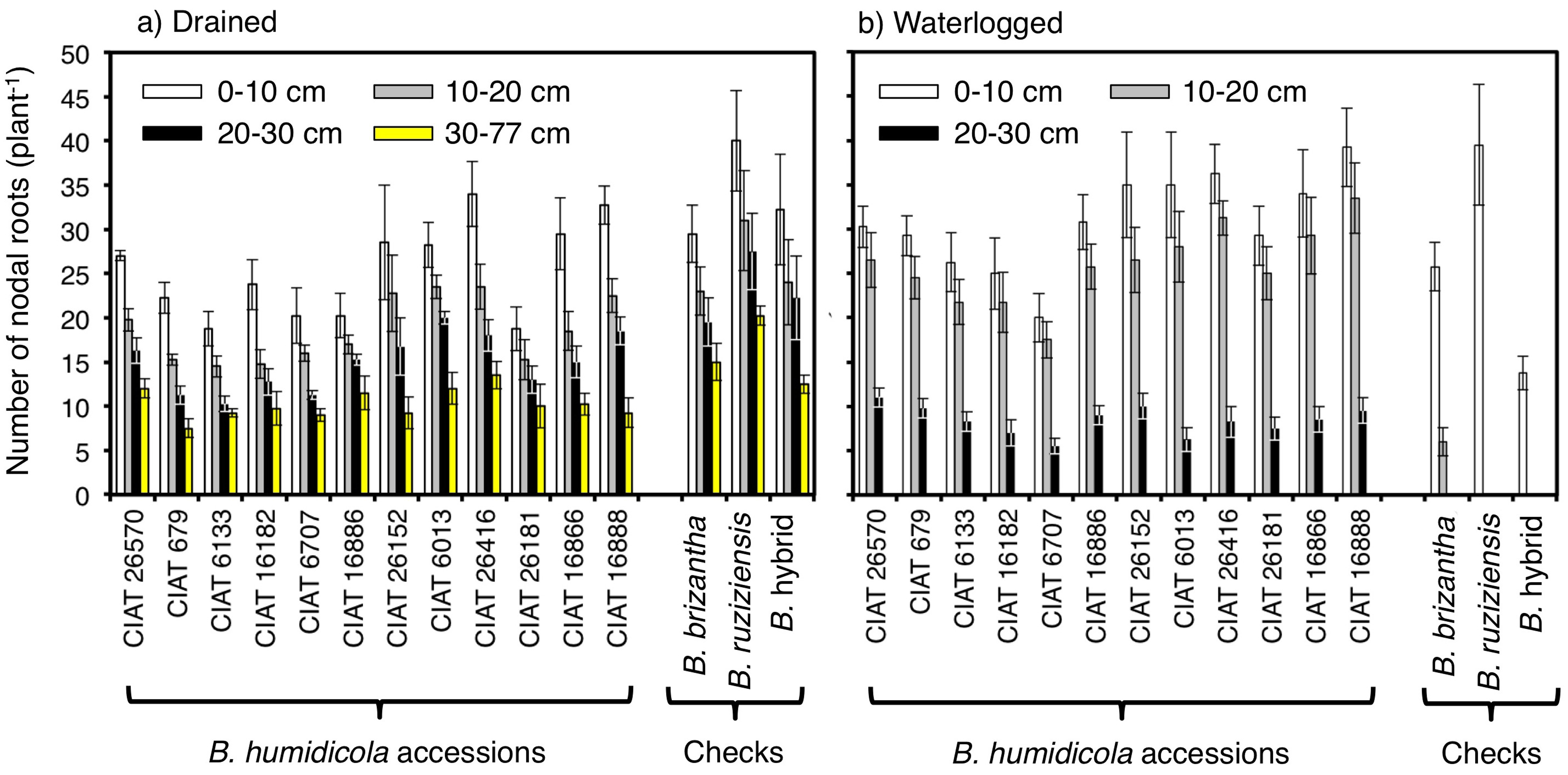

Supplement: Additional Information [file supp_plu017_plu017supp_fig2.doc]
